# Therapeutic strategies against hDOT1L as a potential drug target in MLL-rearranged leukemias

**DOI:** 10.1186/s13148-020-00860-2

**Published:** 2020-05-25

**Authors:** Shahid Banday, Zeenat Farooq, Shabir Ahmad Ganai, Mohammad Altaf

**Affiliations:** 1 Chromatin and Epigenetics Lab, Department of Biotechnology, University of Kashmir, Hazratbal, Srinagar, 190006 India; 2 Present Address: Division of Basic Sciences and Humanities, Faculty of Agriculture, SKUAST-Kashmir, Wadura, Sopore, Jammu and Kashmir 193201 India; 3 Centre for Interdisciplinary Research and Innovations, University of Kashmir, Hazratbal, Srinagar, 190006 India

**Keywords:** Chromatin, Histone methyltransferases, Histone methylation, DNA repair, Mixed lineage leukemia, Gene expression

## Abstract

Therapeutic intervention of proteins participating in chromatin-mediated signaling with small-molecules is a novel option to reprogram expression networks for restraining disease states. Protein methyltransferases form the prominent family of such proteins regulating gene expression via epigenetic mechanisms thereby representing novel targets for pharmacological intervention. Disruptor of telomeric silencing, hDot1L is the only non-SET domain containing histone methyltransferase that methylates histone H3 at lysine 79. H3K79 methylation mediated by hDot1L plays a crucial role in mixed lineage leukemia (MLL) pathosis. MLL fusion protein mediated mistargeting of DOT1L to aberrant gene locations results in ectopic H3K79 methylation culminating in aberrant expression of leukemogenic genes like HOXA9 and MEIS1. hDOT1L has thus been proposed as a potential target for therapeutic intervention in MLL. This review presents the general overview of hDOT1L and its functional role in distinct biological processes. Furthermore, we discuss various therapeutic strategies against hDOT1L as a promising drug target to vanquish therapeutically challenging MLL.

## Introduction

The assembly of eukaryotic genome into a highly complex nucleoprotein structure, called chromatin, controls all DNA-mediated functions of the cell. The fundamental building block of chromatin is the nucleosome core particle, which contains 146 bp of DNA wrapped around an octamer of four core histones namely H2A, H2B, H3, and H4 [1, 2]. The packaging of eukaryotic DNA into chromatin not only facilitates the accommodation of large eukaryotic genome in the small nuclear space, but also blocks access of various enzymes and factors that facilitate DNA-mediated processes like transcription, repair replication and recombination. Several mechanisms operate within the cell to facilitate access to DNA, these include (i) sliding/eviction of nucleosomes by ATP dependent chromatin remodelers, (ii) modifications of protruding histone tails, and (iii) incorporation of variant histones [3–6]. Histone proteins that form an integral part of chromatin contain two domains, a globular domain responsible for histone–histone interactions and a highly dynamic N-terminal tail rich in basic amino acids. Histone tails undergo a number of post-translational modifications which including methylation, acetylation, phosphorylation, ubiquitylation, and ribosylation. These histone modifications either directly alter the chromatin structure or they can serve as binding sites for various transacting factors, which in turn elicit changes in the structure and functionality of the chromatin fiber [1, 3, 4, 7–12]. Histone methylation among the various post-translational modifications has attained lot of focus in recent times due to its role in diverse biological processes that include transcriptional regulation, heterochromatin formation, X-chromosome inactivation, DNA repair, and cellular differentiation. Methylation of histones is mediated by SET-domain containing histone methyltransferases (HMTs), which catalyze the transfer of methyl group from S-adenosy l methionine (SAM) to the ε-amino groups of lysine residues. Further complexity is added by the fact that each lysine residue can accept one, two or even three methyl groups dictating different biological readouts [7, 8]. One exception to SET-domain containing histone methyltransferases is the disruptor of telomeric silencing-1 (hDot1L), which methylates H3K79 positioned within the globular domain of histone H3. hDot1L does not contain a SET domain and its target site is H3K79 located within the globular domain of H3 [13]. hDot1L and its homologous have been implicated to play significant roles in various biological processes like embryonic development, transcription, DNA repair, telomeric silencing, cell-cycle checkpoint, and cardiac function [8, 12]. In this review, we will focus on recent findings regarding histone H3K79 methylation function. Furthermore, we delineate the importance of hDOT1L as a novel therapeutic target in MLL-rearranged leukemias, where mistargeting of hDot1L plays a crucial role in leukemogenesis.

## Dot1 as H3K79 methyltransferase

hDot1L (disruptor of telomeric silencing) also known as lysine methyltransferase 4, (KMT4) is an evolutionarily conserved, sole non-SET domain containing histone methyltransferase, which methylates lysine 79 of histone H3. The homologous counterpart in mammals is called Dot1-like protein (hDOT1L) and shows similar enzymatic properties [13]. hDot1L was initially discovered in *Saccharomyces cerevisiae* where its over-expression disrupts silencing at telomeres [14]. H3K79 methylation is highly conserved across eukaryotes. In *Saccharomyces cerevisiae*, bulk of chromatin is methylated at H3K79. However, regions packaged into heterochromatin lack H3K79 methylation, indicating that hypomethylation of H3K79 is required for proper silencing of heterochromatin [15–19]. Furthermore, mono-, di-, and trimethylation of H3K79 has been shown to block binding of silencing protein Sir3 to chromatin [20]. H3K79 methylation thus acts as a boundary to prevent the spreading of the SIR complex to active regions of the genome. Histone crosstalk has been shown to affect levels of H3K79 methylation. In yeast, monoubiquitination of histone H2BK123 and H2BK120 in mammals is prerequisite for H3K79 methylation. In *Saccharomyces cerevisiae*, deletion of Rad6 (ubiquitin conjugating enzyme) or mutation of H2B123K-A results in complete loss of H3K79 methylation. On the other hand, deletion of hDot1L does not impact H2B-K123 ubiquitination levels [21–23]. Another histone crosstalk that regulates H3K79 methylation in *Saccharomyces cerevisiae* is the histone H4 N-terminal tail. hDot1L binds to a short basic patch on histone H4 tail and this interaction is necessary for methylation of H3K79. On the other side silencing protein Sir3 also binds to H4 N-terminal tail to establish silencing. It has been shown that H4K16 acetylation/deacetylation switch regulates the binding of hDot1L and Sir3 to H4 tail. Thus, in heterochromatin, where H4K16 is deacetylated, Sir3 is a potent inhibitor of hDot1L binding. However, in euchromatin, in which H4K16 is acetylated, Dot1 binds to H4 tail and methylates histone H3K79. So, the weak binding of Sir3 to acetylated H4K16 in euchromatin favors Dot1 binding to H4 tail and subsequent methylation of H3K79 that further blocks Sir3 interaction [20, 24, 25].

## Biological functions of Dot1/H3K79 methylation

Studies in many organisms have linked hDot1L with various biological processes like transcription elongation, DNA damage response, cell cycle regulation, cellular development, and telomeric silencing [12]. In *Saccharomyces cerevisiae, Drosophila* and in human H3K79me2/me3 methylation show strong correlation with transcriptional gene activity [26–28]. Deletions or mutations in *grappa,* hDot1L ortholog in *Drosophila* shows *polycomb* and *tri-thorax group* phenotypes, suggesting a role of H3K79 methylation in developmentally regulated gene expression [29]. H3K79 methylation has been shown to play a crucial role in DNA repair. It acts as a binding site for recruitment of p53-binding protein 1 (53BP1) and its yeast homolog Rad9 to DNA damage sites in vivo. 53BP1 and ScRad9 bind to methylated H3K79 through methyl-lysine-binding Tudor domain and deletion of hDot1L or Tudor domain in ScRad9/53BP1 abolish the recruitment of ScRad9/53BP1 to damages sites and result in a defect in activation of the central checkpoint kinase Rad53 [30–35]. Methylation of H3K79 also plays a role in repair of damaged DNA in G2 phase of cell cycle through Rad9 recruitment, This regulates resection to limit the amount of ssDNA produced during non-homologous end joining (NHEJ) [36, 37].

Recently, H3K79 methylation has been shown to play a crucial role in cell cycle regulation as knockdown of hDot1L results in arrest in G1 phase of cell cycle. It has been observed that H3K79 methylation levels change during cell cycle. In *S. cerevisiae* H3K79me3 methylation does not change during the cell cycle, however the level of dimethyl-H3K79 increase from G1 to S phase and further at G2/M phase [31, 32]. Similarly, in *Trypanosoma brucei,* H3K76me3 levels remain unchanged during the cell cycle whereas H3K76me1 and H3K76me2 are detectable only in G2/M and M phases respectively (in *T.brucei,* two hDot1L homologs Dot1A and Dot1B catalyze methylation of H3K76). Furthermore, deletion of DOT1A causes hypoploidy and replication inhibition whereas its over-expression leads to hyperploidy [38, 39]. In mice, trimethylation of H3K79 is not detectable either in interphase or M phase before the pre-implantation phase of blastocyst stage; however, H3K79me2 is detected at both stages and play a crucial role in spermatogenesis and oocyte development [40, 41]. In human cancer cells like HeLa H3K79me2, levels peak at G1/S transition and then declines towards G2/M phase. Deletion of Dot1 or absence of H3K79 methylation in human cancer cells like HCT116 results in aneuploidy (hypo and hyperploidy), apoptosis, and deposition of non-dividing cells in S phase [42, 43].

One of the earliest functions attributed to hDot1L is its role in telomeric silencing in budding yeast, as overexpression of hDot1L causes silencing defects at telomeres. Furthermore, deletion of hDot1L or mutation of H3K79 causes defect in telomeric silencing by mislocalisation of SIR protein complex [15–18, 44]. *Dot1L-*deficient embryos show several developmental abnormalities like growth impairment, cardiac dilation, blood vessel formation defects in yolk sac, and even death in murine models. Embryonic stem cells (hDot1L-deficient) exhibit H3K79 methylation loss globally besides showing reduction in heterochromatic marks at telomeres and centromeres suggesting the importance of hDot1L and subsequent H3K79 methylation in heterochromatinsation and embryonic development [45].

## hDot1L as a therapeutic target

**Fig. 1:**
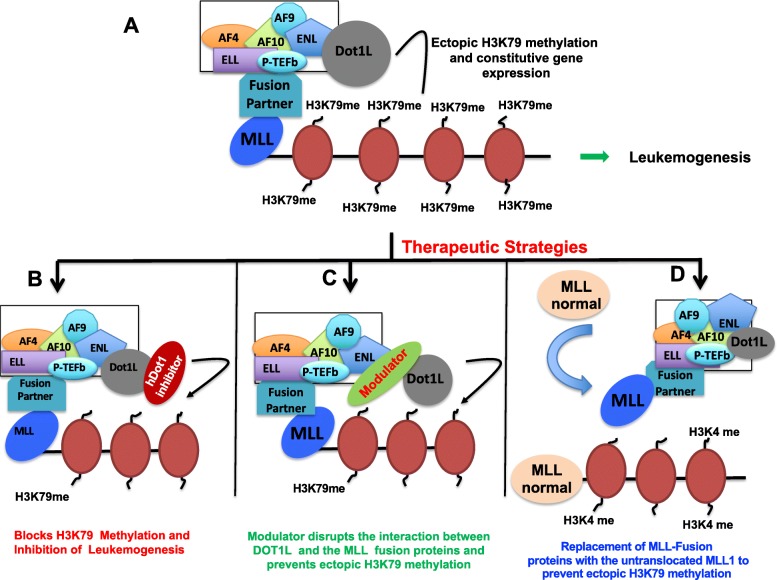
Role of hDot1L in MLL related leukemogenesis. **a** MLL gene, which normally codes for H3K4 methyltransferase, undergoes chromosomal translocations and gets fused to more than 50 recurrent MLL translocation partners. These fusion partners interact with hDot1L and lead to mistargeting of hDot1L to MLL target genes like Hox gene cluster. Ectopic H3K79 methylation and the subsequent activation of the target genes results in leukemogenesis. **b** Small molecules that are capable of inhibiting the HMTase activity of would block H3K79 methylation and subsequent inhibition of leukemogenesis. **c** Another therapeutic strategy would be modulators that block the interaction of hDot1L with MLL fusion proteins. This would prevent ectopic H3K79 methylation and may lead to a novel treatment for MLL-related leukemogenesis. **d** Ectopic H3K79 methylation levels could be prevented by increasing the stability of untranslocated MLL1, thereby replacing the MLL-fusion chimeras

One of the major causes of human cancers is the chromosomal translocation, particularly in acute leukemias. Mixed lineage leukemia (MLL) is an aggressive malignancy and constitutes approximately 70% of infant leukemias. A common feature of MLL disease is a chromosomal translocation affecting the MLL gene on chromosome 11q23, as a result, it becomes fused to a number of proteins and leads to misregulation of MLL target genes [46–48]. Normally, MLL gene codes for H3K4 methyltransferase that catalyzes the methylation of lysine 4 of histone H3 (H3K4) at specific gene loci. MLL is recruited to specific genomic loci through interaction with recognition elements outside the catalytic SET domain [49]. In the disease-linked translocations, the catalytic SET domain is lost and the remaining MLL protein is fused to more than 50 recurrent MLL translocation partners including members of AF and ENL family of proteins such as AF4, AF9, AF10, and ENL. These fusion proteins retain the DNA-binding domain of the MLL, which can direct them to MLL target sites on the genome, but lack the carboxyl-terminal SET domain that possess the histone methyltransferase activity. A hallmark of MLL fusion mediated transformation is its ability to upregulate HOX cluster genes, such as HoxA7 and HoxA9 [8, 44, 49–51]. However, it was puzzling to understand the precise mode of action of MLL fusion proteins. How the fusion proteins mediate an increase in histone methylation while having no histone methyltransferase activity. Recent studies have demonstrated that, increase in histone methylation by fusion proteins is their ability to interact with another histone methyltransferase, hDOT1L [52–55]. As a result, translocation products retain gene-specific recognition elements, but also gain the ability to recruit hDOT1L to these locations, which leads to misregulation of MLL target genes like HOX gene cluster [56–58]. The molecular mechanism of myeloid leukemia was recently discovered when it was found that hDot1L interacts with number of MLL fusion partners and leads to aberrant H3K79 methylation and constitutive activation of HOX genes, which results in leukemic transformation [52–58]. Furthermore, hDot1L lacking methyltransferase activity is capable of suppressing growth of MLL-fusion transformed cells [52]. Hence, in the diseased state, hDot1L itself is not genetically altered; its mistargeted enzymatic activity due to chromosomal translocations is the molecular cause behind mixed lineage leukemia. hDot1L is thus proposed as a catalytic driver of leukemogenesis, and its enzymatic activity is critical to pathogenesis in mixed lineage leukemia (Fig. 1a). Therefore, it has been proposed that inhibition of hDot1L enzymatic activity may provide a target for pharmacological intervention in MLL. hDot1L has thus been proposed as a novel therapeutic target in MLL. Several strategies could be adapted to target hDotl1L in mixed lineage leukemia. These are discussed below.

### Strategy 1: direct inhibition of hDot1L enzymatic activity

As discussed, enzymatic activity of hDotl1L drives leukemogenesis; thus, identification of small molecules that are capable of inhibiting the HMTase activity of hDot1L may lead to a novel treatment for leukemia (Fig. 1a). Recently, EPZ004777 was discovered as a potent inhibitor of hDot1L. It has been shown to block H3K79 methylation in vivo and to inhibit leukemogenic gene expression in selectively MLL translocation-bearing cells [59]. Despite the potency and selectivity of EPZ004777 towards hDot1L, its poor pharmacokinetic properties limit its effectiveness for clinical development. Another second generation hDot1L inhibitor, EPZ-5676 (Pinometostat), has been shown to be more potent than EPZ004777 and causes significant tumor regression in rodent subcutaneous xenograft models of MLL1-rearranged leukemia [60]. Though EPZ-5676 shows high efficacy when used in monotherapy against mixed lineage leukemias bearing MLL rearrangements, its therapeutic efficacy is further augmented by using this inhibitor with other standard of care agents for acute leukemias [61]. It acts in a synergistic manner with cytarabine or daunorubicin against MOLM-13 and MV4-11 *MLL*-r cell lines. EPZ-5676 showed synergistic effect even on combination with DNA hypomethylating agents like azacytidine [61]. While the therapeutic intervention with EPZ4777 shows significant decrease in expression of Hoxa7 and Meis1, the combinatorial treatment involving EPZ4777 with potent SIRT1 agonist (SRT1720) further reduced the expression of defined genes [62]. However, it is important to note that potential inhibitors of hDot1L enzymatic activity must be used with caution since hDot1L is the only known H3K79 methyltransferase and its enzymatic activity is required for normal cellular processes like transcription, DNA repair, embryonic development, and cardiac function. These essential functions of hDot1L in multiple cell types suggest that direct inhibition of hDot11L histone methyltransferase activity might be toxic and not the best option as for as therapeutic targeting hDot1L is concerned.

### Strategy 2: disrupting hDot1L and MLL fusion protein interactions using small molecules

Since hDot1L enzymatic activity is not genetically altered in the MLL per se, its mistargeted enzymatic activity is a direct consequence of the chromosomal translocation affecting MLL patients. Further, inhibiting hDot1L enzymatic activity would have severe consequences for normal cellular processes where hDOT1L activity is required. Consequently, development of alternative therapeutic strategies targeting ectopic H3K79 methylation is important and necessary. Small molecule modulators that could disrupt the interaction between hDot1L and the MLL fusion proteins hold an immense promise as a new generation therapeutic strategy for the treatment of MLL fusion-related leukemias. This targeted disruption of hDot1L interactions with MLL fusion proteins would be more selective to leukemia cells compared to universal inhibition of hDot1L enzymatic activity. The small molecules that could disrupt hDOT1L interactions with MLL fusion proteins would keep the enzymatic activity of hDot1L intact without interfering in its normal cellular processes and would provide a novel pharmacologic basis for therapeutic intervention in MLL (Fig. 1c). The best example for such strategy is the disruption of interaction between murine double minute 2 (MDM2/ubiquitin E3 ligase) and p53 by selective and potent small molecule inhibitors of MDM2 namely Nutlins [63–66]. These inhibitors block the p53-binding pocket of MDM2 and thus reinstate p53 activity [63]. It is widely accepted that p53 gene mutations in infant MLL-ALL are rare and thus drugs rescuing p53 from inhibitory mechanisms may prove propitious [67]. RG7112, an orally available Nutlin, has been tested against MLL-ALL xenografts and resulted in expected p53 upregulation, cell cycle blockade, and finally apoptosis. Further, substantial regressions were seen in these xenografts that were further escalated on combinatorial therapeutic approach [67].

### Strategy 3: increasing the stability of endogenous, non-translocated MLL1 to rescue translocation-mediated MLL1 leukemia phenotype

Another strategy of decreasing the levels of ectopic H3K79 methylation from HOX gene cluster in MLL-rearranged leukemias would involve replacement of MLL-fusion chimeras with endogenous, non-translocated MLL1 containing C-terminal H3K4 methyltransferase required for normal cellular proliferation and development (Fig. 1d). This replacement is however hampered by the excessively low stability of endogenous, untranslocated MLL1. Under normal cellular conditions, MLL1, along with a number of other genes like MLL2 and TFIIA are acted upon by an endopeptidase known as Threonine aspartase 1 (Taspase1) [68–70]. Action of taspase1 on MLL1 results in the generation of a 320 KDa N terminal fragment and a 180 KDa C-terminal fragment. This event leads to decrease in stability and chromatin binding of MLL1 but much less is known about the biological significance of this modification. On the other hand, the MLL1 translocation chimeras lack the taspase1 cleavage site and are therefore more stable. A recent elegant study used taspase1 deficient 293T cells containing MLL-AF4 fusion constructs to see if functional MLL1 could displace MLL1 fusion chimeras from HOX gene clusters in childhood Mixed lineage leukemia. The study reported that the occupancy of uncleaved MLL1 at observed target loci increased substantially. This indicates that functional, uncleaved MLL1 can faithfully bind to MLL1 target genes and displace MLL1 oncogenic fusion products in leukemia background to alleviate some of the symptoms [71]. Indeed, loss of taspase 1 has been shown previously to impede cancer cell proliferation and tumor progression in breast cancer [72–74]. Taspase1, infact, represents a desired therapeutic target for a variety of cancers due to its overexpression in solid and liquid malignancies [75]. Pharmacological inhibition of taspase 1 enzymatic activity using small molecular modulators like NSC48300 would be an attractive option towards stabilizing MLL1 since neither the global H3K4 methylation nor the stability of COMPASS complex are affected by TASP1 gene knock out [71]. However, one of the challenges for adopting this approach is the unique structure and extremely high enzymatic efficiency of taspase 1 which makes its inhibition fairly challenging. In fact, it has been observed that knock down of taspase1 by 70% has not been able to reduce MLL1 cleavage [71]. One of the pre-requisites for taspase1-mediated cleavage of MLL1 is phosphorylation of serine and threonine residues within a consensus patch at a site proximal to taspase1 cleavage site. This phosphorylation event is catalyzed by casein kinase II (CKII). In an attempt to increase the stability of MLL1, it has been seen that inhibition of CKII activity through knock down of its catalytic subunit in HEK-293T cells results in a concomitant increase in the levels of full-length MLL1. Use of CKII inhibitors like CX-4945 or TTP22 also resulted in increased MLL1 full length protein levels and increase in its global occupancy in HEK-293T cells [71–75]. Treatment of leukemia cell lines like MV4-11 (containing MLL-AF4 fusion version) with CX-4945 also displayed an increase in the stability and chromatin occupancy of untranslocated, full-length MLL1. This study has also shown that normal MLL1 version effectively displaces the abnormal translocation version of this protein. It has also been found that treatment of CKII with CX-4945 regresses the size of leukemia in MLL-AF4 mice models [71].

### Strategy 4: hDot1L inhibitors in combinatorial therapy

**Fig. 2:**
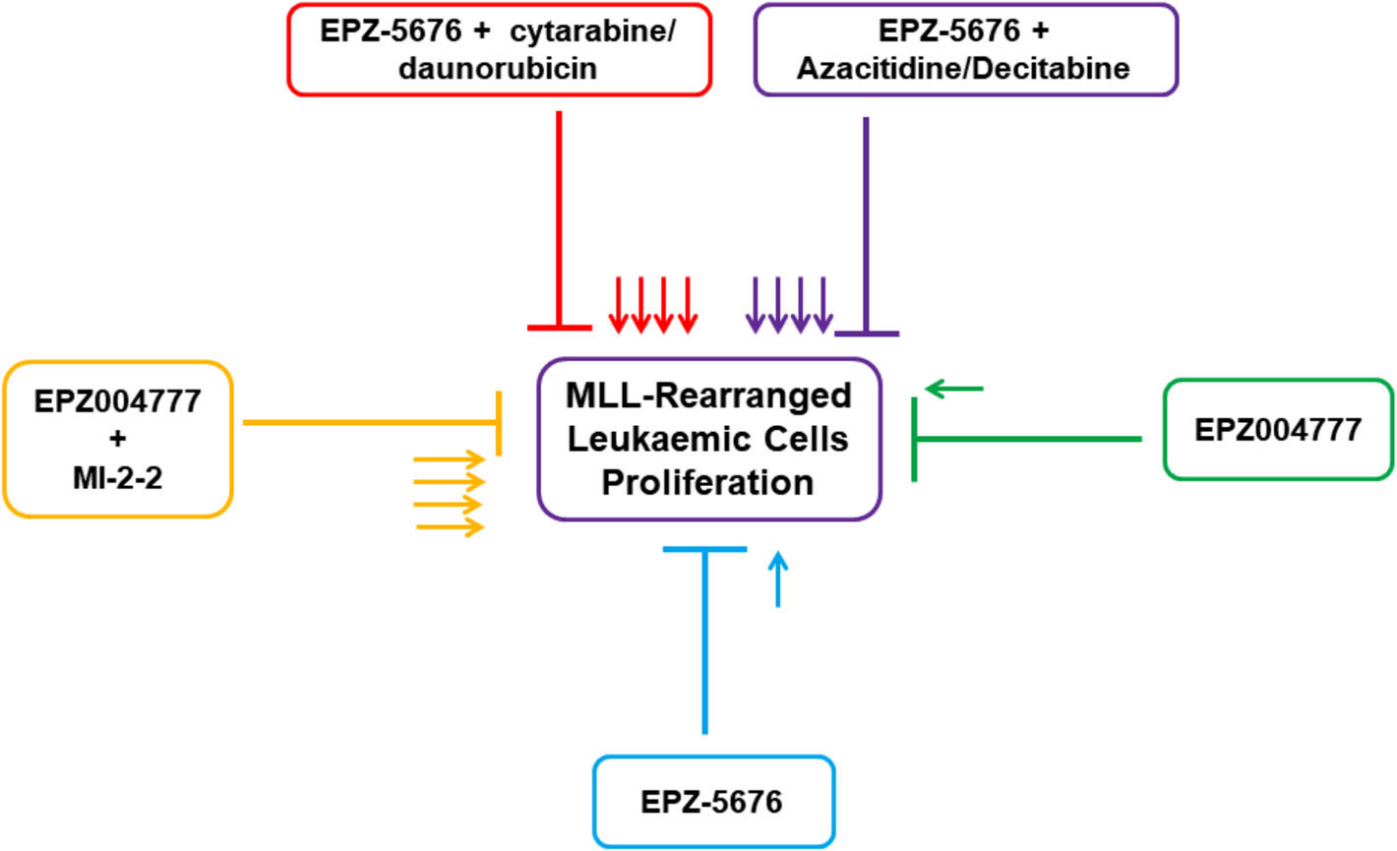
Combination of hDot1L inhibitors with other agents. EPZ-5676 (DOT1L inhibitor) in conjunction with standard of care drugs for AML (cytarabine and daunorubicin) and DNA hypomethylating agents (azacitidine and decitabine) showed synergistic antiproliferative effect against MLL-rearranged leukemic cells. Monotherapy involving only hDot1L inhibitors showed only modest effect as indicated by single arrows in case of EPZ-5676 and EPZ004777. Moreover, another inhibitor of hDot1L (EPZ004777) in combination with MLL-Menin interaction inhibitor (MI-2-2) also showed synergistic growth inhibitory effects in the MLL-rearranged leukemias. The synergistic therapeutic effect is denoted by small multiple arrows in cases involving combined treatment

The limited therapeutic efficacy of hDot1L inhibitors against MLL has been circumvented to a greater extent by using combinatorial therapeutic approaches [76]. The advantage of such approaches is that they mitigate drug resistance markedly and escalated therapeutic effect is achieved even under low-dose combinations [77–79]. Thus, it is quite obvious that conjugated therapies may vanquish dose-limiting toxicity, a major issue in pharmacological intervention [80]. Epigenetically based combinatorial strategies have been tested against MLL-rearranged leukemias [81]. hDot1L inhibitor and EPZ-5676 showed synergistic antiproliferative effect specific against MLL-rearranged leukemic cell lines (MOLM-13 (MLL-AF9) and MV4-11 (MLL-AF4) in conjunction with cytarabine or daunorubicin. The intensity of synergistic effect was found to be sequence dependent as simultaneous treatment with defined agents showed relatively better efficacy than sequential mode [61]. EPZ-5676 also showed synergistic effect with DNA methyltransferase inhibitors (azacitidine and decitabine). This synergy was also MLL-rearranged leukemia-specific [61]. Clinical studies for assessing the tolerability and initial efficacy of combined therapy involving EPZ-5676 and azacitidine as therapeutic agents are currently being conducted on R/R *MLL*-R AML patients [82]. Another hDot1L inhibitor EPZ004777 has been used in combination with MLL-Menin interaction inhibitor (MI-2-2). This strategy markedly enhanced induction of differentiation and cytotoxicity in various MLL disease models. The combined treatment expectedly repressed the MLL and MYC target genes in leukemic cells [83]. One of the key events that happen due to aberrant expression of HOX genes by mistargeting of fusion proteins is induction of differentiation blockade, which results in leukemic cells with stem cell-like properties and increased growth and survival rates. This differentiation blockade is an important mechanism for MLL fusion-mediated properties. Differentiation therapy is emerging as an important therapeutic option for MLL fusion related mediated leukemias. A recent study used clinical stage inhibitors for BET, DHODH, hDot1L, and inhibitors for CDK9 and the Menin-MLL interaction with a focus on differentiation induction either alone or in combination. It is reported that Menin-MLL and hDot1L inhibitors are specific to MLL-fused leukemia cell lines, whereas inhibitors of BET, DHODH, and P-TEFb have work beyond leukemic cell lines. Further, differentiation induction was observed for Menin-MLL, DOT1L, and DHODH inhibitors. In addition, synergistic impact on differentiation induction was observed when BAY-155, Brequinar, and EPZ-5676 were combined [84]. In addition, MLL-fusion proteins have been shown to form complexes with epigenetic reader BRD4, polymerase-associated factor complex (PAFc), and super elongation complex (SEC) to sustain the expression of oncogenes in MLL-rearranged leukemias [85, 86]. Dot1L-mediated methylation of H3K79 opens up the chromatin and facilitates the recruitment of histone acetyltransferase p300 which then recruits BRD4. Further, hDot1L and DNA methyltransferase and DNMT3A in coordination are also associated with MLL-rearranged leukemias. Loss of DNMT3A leads to increase in H3K79 methylation in hematopoietic cell systems [86]. These studies suggest that the use of hDot1L inhibitors in combination with other small molecules targeting other proteins hold immense promise in anticancer therapy against MLL-rearranged leukemias (Fig. 2).

## Conclusions and perspectives

The important role for hDot1L in MLL-rearranged leukemias is well established because a critical step in transformation and disease progression in MLL is the aberrant recruitment of hDot1L L by MLL fusion proteins. This is strongly supported by functional evidence demonstrating that a loss of hDot1L HMTase activity profoundly affects the leukemogenic gene expression program and leukemogenic activity of *MLL* fusion-transformed cells. The development of small molecule inhibitors for hDot1L is clearly warranted because such molecules will help to address some of the outstanding mechanistic questions, and most importantly, they may change the outlook for patients with this devastating type of leukemia that carries a poor prognosis. Small-molecule inhibitors of h hDot1L have shown encouraging results in various cellular and xenograft models of MLL. These inhibitors have been found to suppress the expression of genes (*Hoxa7* and *Meis1*) involved in promoting and maintenance of vicious MLL. These inhibitors exhibit selective antiproliferative effect against (MLL-r) cell models, which determines their safety and clinical relevance. EPZ-5676 has shown acceptable toxicity profile during phase I clinical trials. While these inhibitors show high efficacy as single agents, the combined therapeutic regimens involving these inhibitors in conjunction with other agents like cytarabine, daunorubicin, DNA hypomethylating agents, and SIRT1 activators further enhances their therapeutic efficacy. Despite the remarkable efforts for finding the active site-based hDot1L inhibitors for pharmacological intervention against MLL, no efforts have been taken to find novel inhibitors targeting the hDot1L sites critical for its interaction with MLL fusion proteins. In such scenarios, the HMTase activity of hDot1L would remain unaffected, which is required for normal activities of the cell, but at the same time would prevent its association with MLL fusion partners. Furthermore, this targeted disruption of hDot1L interactions with MLL fusion proteins would be more selective to leukemia cells compared to universal inhibition of hDot1L enzymatic activity. Another novel strategy would be replacement of MLL1 fusion chimeras with endogenous, non-translocated MLL1 by increasing the stability of untranslocated MLL1 using targeted small molecules against the proteases. Since in clinical trials, the hDot1L inhibitors in monotherapy are showing modest efficacy in treating MLL-rearranged leukemias; therefore, combinatorial approach targeting different proteins would offer better therapeutic response against MLL-rearranged leukemias.

## Data Availability

Not applicable.

## Abbreviations

hDot1L: Disruptor of telomeric silencing
MLL: Mixed lineage leukemia
HMTs: Histone methyltransferases
SAM: S-adenosy l methionine
KMT4: Lysine methyltransferase 4
53BP: p53-binding protein 1
CKII: Casein kinase II
NHEJ: Non-homologous end joining
